# A Long-Term Assessment of the Variability in Winter Use of Dense Conifer Cover by Female White-Tailed Deer

**DOI:** 10.1371/journal.pone.0065368

**Published:** 2013-06-13

**Authors:** Glenn D. DelGiudice, John R. Fieberg, Barry A. Sampson

**Affiliations:** 1 Forest Wildlife Populations and Research Group, Minnesota Department of Natural Resources, Forest Lake, Minnesota, United States of America; 2 Department of Fisheries, Wildlife, and Conservation Biology, University of Minnesota, St. Paul, Minnesota, United States of America; 3 Biometrics Unit, Minnesota Department of Natural Resources, Forest Lake, Minnesota, United States of America; 4 Forest Wildlife Populations and Research Group, Minnesota Department of Natural Resources, Grand Rapids, Minnesota, United States of America; Bangor University, United Kingdom

## Abstract

**Backgound:**

Long-term studies allow capture of a wide breadth of environmental variability and a broader context within which to maximize our understanding of relationships to specific aspects of wildlife behavior. The goal of our study was to improve our understanding of the biological value of dense conifer cover to deer on winter range relative to snow depth and ambient temperature.

**Methodology/Principal Findings:**

We examined variation among deer in their use of dense conifer cover during a 12-year study period as potentially influenced by winter severity and cover availability. Female deer were fitted with a mixture of very high frequency (VHF, *n* = 267) and Global Positioning System (GPS, *n* = 24) collars for monitoring use of specific cover types at the population and individual levels, respectively. We developed habitat composites for four study sites. We fit multinomial response models to VHF (daytime) data to describe population-level use patterns as a function of snow depth, ambient temperature, and cover availability. To develop alternative hypotheses regarding expected spatio-temporal patterns in the use of dense conifer cover, we considered two sets of competing sub-hypotheses. The first set addressed whether or not dense conifer cover was limiting on the four study sites. The second set considered four alternative sub-hypotheses regarding the potential influence of snow depth and ambient temperature on space use patterns. Deer use of dense conifer cover increased the most with increasing snow depth and most abruptly on the two sites where it was most available, suggestive of an energy conservation strategy. Deer use of dense cover decreased the most with decreasing temperatures on the sites where it was most available. At all four sites deer made greater daytime use (55 to >80% probability of use) of open vegetation types at the lowest daily minimum temperatures indicating the importance of thermal benefits afforded from increased exposure to solar radiation. Date-time plots of GPS data (24 hr) allowed us to explore individual diurnal and seasonal patterns of habitat use relative to changes in snow depth. There was significant among-animal variability in their propensity to be found in three density classes of conifer cover and other open types, but little difference between diurnal and nocturnal patterns of habitat use.

**Conclusions/Significance:**

Consistent with our findings reported elsewhere that snow depth has a greater impact on deer survival than ambient temperature, herein our population-level results highlight the importance of dense conifer cover as snow shelter rather than thermal cover. Collectively, our findings suggest that maximizing availability of dense conifer cover in an energetically beneficial arrangement with quality feeding sites should be a prominent component of habitat management for deer.

## Introduction

For at least 60 years, wildlife researchers and managers have been describing the prevalence of dense conifer stands and their use by northern white-tailed deer (*Odocoileus virginianus*) on winter ranges [1]–[6] and documenting the negative impacts that winter weather conditions have on deer survival and reproduction [7]–[10]. These impacts have been related to nutritional restriction and poor condition, predation, or a combination of the two [10]–[15].

Given the potential effects of winter severity on population performance of northern deer species, studies have focused on the weather-moderating attributes of dense conifer stands, specifically assessing their potential value as thermal cover [16]–[19]. However, there is little evidence from these studies or others conducted under controlled conditions that the potential energetic benefits of thermal cover actually translate to improved winter condition, reproduction, or survival of deer or other cervids [20]–[22].

The potential value of dense conifer stands as snow shelter for deer in the northern Great Lakes region becomes particularly evident when snow cover accumulates to depths that physically impede their mobility, markedly increase energetic costs of movement, and decrease browse availability [4], [18], [23]–[24]. Snow depths of ≥40 cm seriously restrict movements of white-tailed deer [24]–[27], but depths within conifer stands may be reduced by as much as 36 percent due to interception of snowfall by canopies ≥70 percent [16], [28]. Increasing snow depths have been directly related to increased wolf predation [12]–[13] and reduced overall winter survival, whereas no such relationships between ambient temperatures and survival were detected [9]–[10], [29].

It is unclear whether minimum ambient temperature or deepening snow cover has the most pronounced effect on deer use of dense conifer cover. A number of studies have indicated that low temperatures and cold winds (or air chill) may have the greatest impact on prompting deer to migrate to winter yards (high concentration areas) with shelter, whereas movements within those areas and use of dense cover specifically may be most strongly influenced by increasing snow depths [4], [26], [30]–[31]. Others have questioned the “need” for thermal cover when available nutrition is adequate to fulfill energetic requirements [17], [21], [24], but even when it is not, the work of Cook et al. [21] suggests that the weather-moderating influences of conifer cover may be too small, infrequent, and variable to convey biologically significant benefits. Actually, the thermal benefits afforded to free-ranging cervids from increased daytime exposure to solar radiation in open areas are likely of greater relative value to their energetic balance and fitness than the potential thermal benefits associated with dense cover, particularly when ambient temperatures are coldest [3], [21], [32].

Given the wide variation of periodicity, intensity, and duration of climatic factors, such as ambient temperature and snowfall, winter severity and its effect on deer behavior can be highly variable from year to year [33]. Long-lived species like white-tailed deer have been naturally selected to withstand such variability, and therefore are of central interest in studies of environmentally-induced behavioral responses. Long-term studies provide the opportunity to capture a wide breadth of environmental variability and a broader context within which to examine and maximize our understanding of relationships to specific aspects of wildlife behavior [34]–[35].

Our long-term study in north-central Minnesota (MN), USA, was prompted by a management concern for increasing deer densities relative to available dense conifer cover. The goal of our study was to improve our understanding of the biological value of dense conifer cover to deer on winter range. During a 16-year period, winter severity varied widely, as did its effect on autumn migration of radiocollared female deer to winter range and survival [29], [36]–[37]. In addition to capturing a wide range of winter weather conditions, we collected data on four study sites that varied in their availability of conifer cover. To develop alternative hypotheses regarding expected spatio-temporal patterns in the use of dense conifer cover (or open cover types), we considered two sets of competing sub-hypotheses. In the first set, we considered the following 2 possibilities: 1a) dense conifer cover is not limiting on any of our four study sites, versus 1b) dense conifer cover is limiting on one or more of the sites. In the second set, we considered four alternative hypotheses regarding the potential influence of snow depth and ambient temperature on space-use patterns, specifically: 2a) the use of dense conifer cover will increase as snow depth increases, 2b) the use of dense conifer cover will decrease with decreasing daytime temperature as individuals take advantage of thermal benefits of open habitat, 2c) the use of dense conifer cover will increase with increasing snow depth, but decrease with decreasing daytime temperature as use of open habitat increases; and 2d) the use of conifer cover will be relatively constant with respect to changes in snow depth and daytime temperatures.

The combination of these two sets of sub-hypotheses gives rise to 8 alternative expected space-use patterns (Table 1). We evaluated relative support for our alternative hypotheses by comparing temporal patterns in the use of dense conifer cover across the four study sites relative to changes in temperature and snow depth. We monitored deer use of dense conifer cover and open habitat by a mixture of very high frequency (VHF) telemetry and Global Positioning System (GPS) collar technology, each with advantages and disadvantages. Use of VHF telemetry from fixed-wing aircraft involved more individual deer, covered more years and more variable winter weather conditions (i.e., mild to severe), but locations were collected less frequently than with GPS technology (1/hr or 1/4 hr). Thus, in addition to exploring spatio-temporal patterns in the use of conifer cover and open habitat, we also discuss the implications of the two data collection methods relative to our ability to learn about how environmental variability impacts habitat use, other behavioral responses, and ultimately, fitness.

**Table 1:** Description of eight alternative expected space-use patterns of adult (≥1.5 years old) female white-tailed deer relative to dense conifer cover, derived from the combination of two sets of sub-hypotheses, one addressing availability of dense conifer cover on four study sites and the other their response to increasing snow depth or decreasing ambient temperature, in north-central Minnesota, 1 November–14 May 1993–1994 to 2004–2005.

1A2A	We expected to see similar trends on all 4 sites, with the use of dense conifer increasing significantly as snow depths increase (with a potential nonlinear response around 40 cm).
1B2A	We expected to see a more significant response of increased use of dense conifer cover with increasing snow depth on sites with more cover.
1A2B	We expected to see similar trends on all 4 sites, with decreased use of dense conifer stands (i.e., increased use of open habitat) with decreasing daytime temperatures.
1B2B	We expected to see a more significant response of decreased use of dense conifer cover with decreasing daytime temperatures on sites with more dense cover.
1A2C	We expected to see similar trends on all 4 sites, with increased use of dense conifer cover with increasing snow depth and decreased use with decreasing daytime temperatures.
1B2C	We expected to see a more significant response of increased use of dense conifer cover with increasing snow depth and decreased use with decreasing temperature on sites with more cover.
1A2D	We expected the use of dense conifer cover to be similar at all 4 sites and relatively constant with respect to snow depth and minimum daytime temperature.
1B2D	We expected to see higher use of dense conifer cover on sites with more cover, but a relatively constant response with respect to snow depth and minimum daytime temperature.

## Methods

### Study Area

Our study included four winter range sites located along the southeastern boundary of the Chippewa National Forest in north-central MN (46°52′–47°15′N and 93°45′–94°07′W). The Willow (Wil), Inguadona (Ing), Shingle Mill (Shi), and Dirty Nose (Dir) sites were 20, 24, 23, and 13 km^2^, respectively. Topography is undulant with elevations ranging from 400 to 475 m above sea level. The uplands were dominated by deciduous and mixed deciduous-conifer stands, which included trembling aspen (*Populus tremuloides*), balsam poplar (*Populus balsamifera*), paper birch (*Betula papyrifera*), black ash (*Fraxinus nigra*), balsam fir (*Abies balsamea*), red pine (*Pinus resinosa*), and jack pine (*Pinus banksiana*) [38]. Northern white cedar (*Thuja occidentalis*), black spruce (*Picea mariana*), balsam fir, and tamarack (*Larix laricina*) were most apparent on the lowlands. Although the winter diet of deer on the four sites was highly diverse (about 36 browse species), beaked hazel (*Corylus cornuta*), mountain maple (*Acer spicatum*), and red-osier dogwood (*Cornus stolonifera*) accounted for 82 and 89% of species browsed during mild and severe winters, respectively [39].

We calculated a Minnesota Department of Natural Resources (MNDNR) winter severity index (WSI) by accumulating 1 point for each day with a snow depth ≥38 cm and 1 point for each day with an ambient temperature ≤ −17.7°C during November-May. During winters 1990–1991 to 2004–2005, maximum WSIs ranged from 45 to 195, snow depths ranged from 0 to 98 cm, and were associated with mortality rates of 0.06 and 0.37, respectively [29]. Monthly mean daily minimum and maximum temperatures (between November and May) ranged from −28° to 6°C and −15° to 24°C, respectively [40]. During 1971–2000, the mean annual snowfall was 134 cm, and the mean temperature for January (coldest month) was −13.5°C [40].

Sixty-eight percent of 335 radiocollared female deer were classified as seasonal migrators, inhabiting spatially non-overlapping winter and spring-summer-fall home ranges during this long-term study [37]. Migration distances ranged from 1.5 to 34.8 km; annual means ranged from 9.4 to 14.7 km.

Wolf (*Canis lupus*) predation is the primary source of natural mortality of adult deer in north-central and northeastern MN [9]–[10], [12], [29]. Wolf numbers and occupied range in northern MN have been stable since the mid- to late 1990s [41]; the most recent (2008) population estimate was 2,921 wolves. Black bears are a major source of mortality of deer neonates through the summer months [42]–[43]. According to the most accurate point estimates, black bear numbers ranged between approximately 15,000 and 26,000 during 1991 to 2008 [44]–[45].

### Deer Capture, Handling, and Monitoring

We captured deer primarily (95%) by Clover traps [46] during January-March 1991–2005, but augmented these efforts with captures by rocket-net and net-gun deployed from helicopter [36]. Generally, we chemically immobilized blind-folded, physically restrained deer with 75–100 mg of xylazine HCl and 300–400 mg of ketamine HCl injected intramuscularly. We blood-sampled and catheterized females for urine; weighed, ear-tagged, and physically examined them; monitored rectal temperatures; extracted a last incisor for age determination by cementum annuli; and fitted each with either a VHF (Telonics, Inc., Mesa, AZ) or GPS (G-2000, Advanced Telemetry Systems, Inc., Isanti, MN) radiocollar. Details of this handling have been reported elsewhere [36], [47]. We checked pregnancy status in the field by dop-tone ultrasound. We reversed anesthesia with an intravenous injection of 15 mg of yohimbine. On the rare occasion that an injured deer had to be euthanized, it was chemically immobilized as described above and injected intravenously with a saturated solution (300 mg/ml) of potassium chloride (50 mg KCL per kg body weight [48]). Animal capture and handling protocols were approved by the University of Minnesota’s Institutional Animal Care and Use Committee under Animal Subjects Code Numbers 9701A00007, 9911A25961, and 0208A29962 and meet the guidelines recommended by the American Society of Mammologists [49]. The permitting authority was the Minnesota Department of Natural Resources.

During winter (1 November–14 May), we attempted to locate VHF-collared deer from fixed-wing aircraft as many times as possible each week, given the inherent constraints (e.g., weather conditions) associated with this technique [50]–[52]. We followed a total of 267 deer (seasonal migrators and those sedentary on the winter study sites year-around) using VHF technology during winters 1993–1994 to 2004–2005 (Figure S1). Most deer were followed for 1–2 years (mean = 1.8, interquartile range = 1 to 2). The number of locations per deer was highly variable (mean = 20, interquartile range = 3–25, min = 1, max = 144; Figure S2).

During winters 2000–2001, 2001–2002, 2003–2004, and 2004–2005, we deployed GPS collars on a total of 24 deer at least 1.5 years old. We pre-programmed collars to attempt a location either every hour or every four hours, depending on the date, life history events (e.g., fawning), and battery-life considerations. We followed two individuals in winter 2000–2001, four in 2001–2002, 10 in 2003–2004, and nine in 2004–2005. At 1- and 4-hour intervals, the mean fix-rate was 68.9% and 69.7% during 2004 and 84.7 and 85.8% during 2005, respectively.

### Habitat Composition of Sites

We used mirror stereoscopes (Leitz, Forestry Suppliers, Inc., Jackson, MO) and 9″×9″ and 4″×6″ *leaf-off*, color infrared air photos (1∶15, 840”) to delineate and map forest stands according to a classification system used to assign dominant tree species, classes of height (<20′, 20′≤×<35′, and ≥35′) and conifer canopy closure (open [A], <40%; moderately dense [B], 40% ≤×<70%; and dense [C], ≥70%). We also delineated openings and hardwood types. We collected four to eight ground control points (GCP) for each photo using a Trimble Geo-Explorer GPS (Trimble Navigation Limited, Sunnyvale, CA). We collected GCPs by averaging 300 points recorded at each location. These GCPs were then post-processed to improve accuracy using Trimble’s Pathfinder software and a base station file from a station located in Duluth. We digitized our original vector line coverage in EPPL7 (a GIS developed by the Land Management Information Center, Department of Administration, State of MN).

We performed all digitizing using the Universal Transverse Mercator (UTM) Zone 15 North Coordinate System. Habitat polygons were delineated down to a patch size of 0.5 hectare. Once the digital line file was created, it was uploaded into ArcInfo 6.0 (Environmental Systems Research Institute [ESRI], Redlands, CA), and the polygons were cleaned and built. Creating a polygon identifies undershoots and overshoots of a line and snaps them together to ensure that all the polygons are closed. Building a polygon identifies the set of arcs that defines each polygon, the internal number of polygons to the left and right side of each arc in a polygon and also builds a polygon attribute table. Once the polygons were cleaned and built, the polygon attribute table was populated with the forest habitat data from the air photointerpretation.

**Figure 1:**
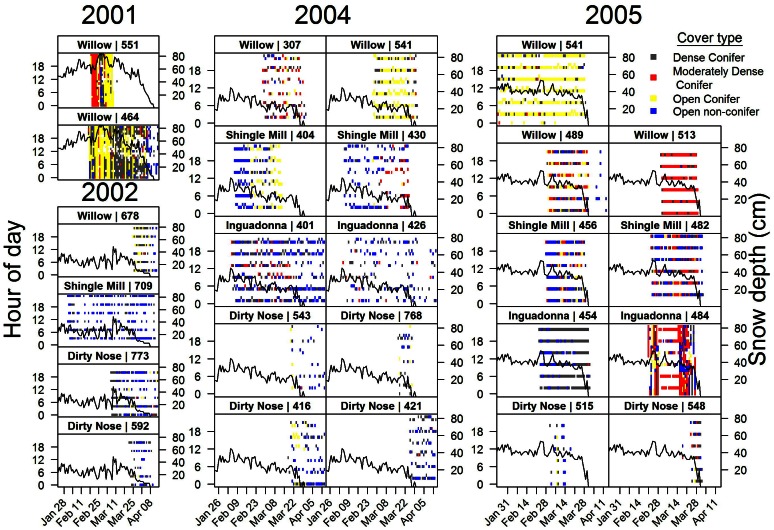
Availability (versus time) of dense (≥70% canopy closure, left panel) and moderately dense (40% ≤×<70% canopy closure, right panel) conifer cover for each of four study sites, north-central Minnesota, 1991–2005. First-year baseline was dependent on the year the site was incorporated into the study and habitat quantified.

During the course of our long-term study, air photo flights were flown annually to capture any timber harvests that occurred on each study site. Once a cut occurred we digitized stand alterations for each site, and the coverage was updated (Figure 1). This process resulted in new coverage for each study site for most years of the study. Beginning in 1999, all cuts were digitized using air photos scanned in high definition and “heads up digitizing” in Arcview 3.3 (ESRI). These photos were rectified on the screen using rectification points plucked from the 1991 U. S. Geological Survey (USGS), National Aerial Photography Program (NAPP) photos.

In 2005, we updated all coverage attribute tables to account for changes in conifer canopy closure class (open, moderate, and dense) which occurred during the study period by year due to forest succession. The difference between the initial and final percent canopy closure class of conifer stands was calculated and divided by the number of years between the air photointerpretations to derive the annual percent change. The year specific conifer stands succeeded to the next canopy closure class was noted. We used ArcGIS (ArcMap Version 9.3.1) to depict and measure areas of the four study sites (km^2^) and forest cover types (ha) within each site, and to overlay winter VHF- and GPS-derived locations of radiocollared deer.

### Data Analyses

#### VHF telemetry

Similar to Kneib et al. [53], we fitted multinomial response models with the following structure:
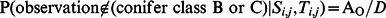


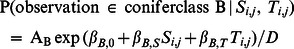


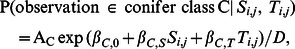
with *S_i,j_* and *T_i,j_* giving the snow depth (cm) and minimum daily temperature (^o^C) measured on day *i* of year *j*, respectively, and 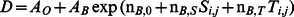 + 
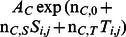
, a normalizing constant that ensures the probabilities sum to 1. The availabilities of moderately dense conifer cover (B), dense conifer cover (C), and ‘other’ habitat (includes open conifer [≤40% canopy closure], openings, and hardwoods), or A_B_, A_C_, and A_O_, respectively, were adjusted yearly to account for timber harvest and succession (Figure 1). The *β*’s quantify the increase in use of moderately dense (B) and dense conifer (C) cover classes relative to the ‘other’ category as a function of snow depth and minimum daily temperature. If all *β*’s are 0, then we recover a null model that assumes use of each habitat type is proportional to its availability.

Rather than use random effects to account for repeated measures and within-animal correlation (as in [53]), we used a generalized estimating equation approach with a ‘working independence assumption’ for inference [54]. Specifically, we constructed an objective function equivalent to the likelihood for independent data and then used the R function ‘optim’ [55] to find regression parameters that maximized this objective function. We accounted for the repeated measures design by using a non-parametric bootstrap, re-sampling individuals with replacement. Thus, we treated the observations as though they arose from a two-stage cluster design, with the first stage representing individual animals on the study site (sampled independently) and the second stage representing locations of these animals [44], [56]. This approach has the advantage of simplicity, but more importantly, the regression parameters reflect population-level response patterns that are of primary interest to managers [57].

#### GPS data

We constructed date-time plots to explore diurnal and seasonal patterns of habitat use, as well as among-individual variability in these patterns. Specifically, for each deer, we constructed a level or image plot with the *x*-axis depicting Julian date (23 January-April 14) and *y*-axis depicting hour of day (0–23), with color used to indicate the cover type associated with each observed location. In addition, we overlaid time series of estimated snow depths and minimum temperature to explore habitat use patterns relative to changes in snow depth and temperature.

## Results

### VHF Data

**Figure 2:**
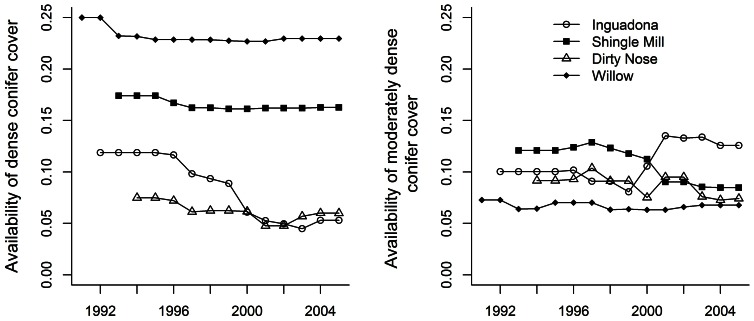
Model-based predicted probabilities of adult (≥1.5 years old), female white-tailed deer using dense (≥70% canopy closure, yellow) and moderately dense (40% ≤×<70% canopy closure, red) conifer cover, and “other” (here includes conifer with <40% canopy closure, openings, and hardwoods; blue) during daytime hours (i.e., 0730–1700 hr) as a function of snow depth (panels A-D) and minimum daily temperature (panels E-H), for each of four study sites, north-central Minnesota, 1 November–14 May 1993–1994 to 2004–2005. Colored bands depict point-wise 95% bootstrap confidence intervals. To generate model-based response curves, we set availabilities of each habitat type to site-specific mean values. Similarly, we set daily snow depths (for bottom panels) and minimum temperatures (for top panels) to site-specific means.

At all four sites deer made greater daytime use of more open vegetation types (open conifer, openings, and hardwoods) compared to moderately dense and dense conifer cover when snow depths were shallow to moderate (<40 cm; Figure 2A–D). Yet, estimates of *β*_C,S_ were positive for the Wil, Shi, Dir, and Ing sites and significantly different from 0 (α = 0.05) at all but Dir (Table 2), suggesting deer increased their use of dense conifer cover as snow depth increased (Figure 2A–D). Further, population responses to increases in snow cover were most pronounced at Wil and Shi, the two sites with the greatest amount of dense conifer cover (Figures 1 and 2). With no snow cover (and minimum daily temperatures and dense conifer availabilities both set to site-specific mean values), the estimated probability of use of dense conifer was ≤0.23 for all four sites (Figure 2A–D); however, at maximum snow depths (90–100 cm) the estimated probability of use of dense conifer was 2–3 times greater at Wil and Shi, respectively, than at Dir and Ing. The estimate of *β*_B,S_ also was positive and significantly different from 0 for Shi (Table 2), suggesting increased use of moderately dense conifers at this site as snow depth increased. Simultaneously, deer use of open types decreased dramatically with increasing snow depths at Wil and Shi.

**Table 2:** Regression parameter estimates (95 percentile-based bootstrap confidence intervals)^1^ from habitat use models fitted to very high frequency (VHF) location data collected from a total of 267 adult (≥1.5 years old), female white-tailed deer, north-central Minnesota, 1 November–14 May 1993–1994 to 2004–2005.

	Study sites			
Parameter^2^	Willow	Dirty Nose	Shingle Mill	Inguadona
*β*_B,0_	1.282 (0.598, 1.827)	−0.197 (−0.878, 0.418)	−0.359 (−0.982, 0.210)	0.999 (0.593, 1.295)
*β*_C,0_	0.520 (0.068, 0.913)	0.992 (0.148, 1.633)	−1.151 (−1.859, −0.462)	0.367 (−0.204, 0.805)
*β*_B,S_ (snow)	0.002 (−0.006, 0.013)	0.011 (−0.0001, 0.020)	**0.023 (0.013, 0.033)**	−0.004 (−0.013, 0.007)
*β*_B,T_ (temp)	0.008 (−0.010, 0.030)	0.016 (−0.013, 0.049)	**0.032 (0.001, 0.066)**	−0.010 (−0.030, 0.013)
*β*_C,S_ (snow)	**0.018 (0.012, 0.025)**	0.003 (−0.010, 0.019)	**0.040 (0.026, 0.055)**	**0.009 (0.001, 0.018)**
*β*_C,T_ (temp)	**0.019 (0.004, 0.032)**	0.013 (−0.013, 0.050)	**0.042 (0.024, 0.063)**	0.002 (−0.014, 0.017)

1 Confidence intervals that do not include 0 are in bold, indicating significance at α = 0.05.

2 B represents the moderately dense (40%≤×≤70%) conifer canopy closure class and C represents the dense (≥70%) conifer canopy closure class.

Estimates of *β*_C,T_ also were positive for all four study sites and significantly different from 0 for Wil and Shi (Table 2), indicating a more significant response of decreased use of dense conifer as daily minimum temperatures decreased on sites with more dense cover (Figure 2E–H). This was associated with a more significant response on these same sites of increased use by deer of open types as temperatures decreased (Figure 2E–H). The estimate of *β*_B,T_ (for moderately dense conifer) also was positive and significantly different from 0 for Shi (Table 2, Figure 2G).

### GPS Data

**Figure 3:**
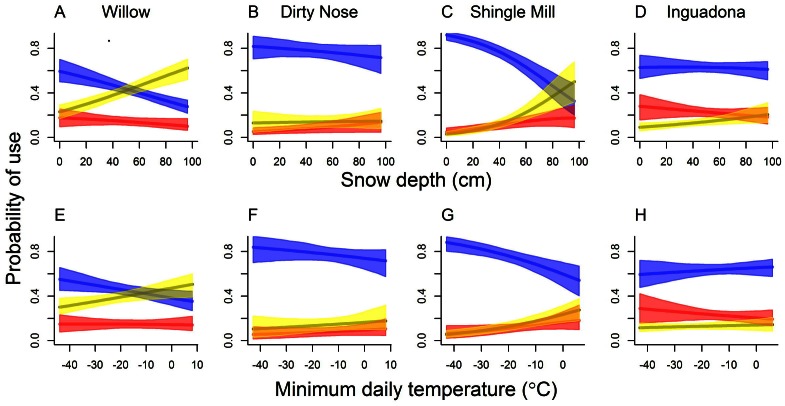
Date-time plots illustrating individual variability in use of dense (≥70% canopy closure), moderately dense (40% ≤×<70% canopy closure), and open conifer cover (<40% canopy closure), and open non-conifer types (openings and hardwood types) by adult (≥1.5 years old), female white-tailed deer monitored using Global Positioning System (GPS) collars collecting locations hourly or every four hours on four study sites, north-central Minnesota, 23 January–14 April 2001, 2002, 2004, and 2005. The solid black line represents average weekly snow depths.

Average snow depths were typically modest (≤40 cm) during 3 of the 4 winters when GPS collars were deployed on deer; however, during winter 2000–2001, snow depth peaked at 80 cm (Figure 3). There was considerable among-animal variability in their propensity to be found in dense, moderately dense, and open conifer stands, or open non-conifer types (“other,” Figure 3). Some individuals were almost always located in a single habitat type. For example, in 2002, Deer 709, 773, and 592 spent a great deal of time in open non-conifer habitats. Similarly, Deer 513 (in 2005) was typically located in moderately dense conifers, and Deer 541 in both 2004 and 2005, was most frequently located in dense conifer, despite the very different snow depths in these winters. During moderately severe winter 2000–2001, the two GPS-collared deer at Wil both made intense use of dense conifers for 2–4 weeks. Some animals used a variety of habitat types, but there was a lot of inertia (i.e., individuals tended to use the same cover type for long periods of time [e.g., see Deer 551 and 464 in 2001]). Any diurnal pattern was relatively weak; animals largely seemed to make similar use of habitat types during the day and night (Figure 3).

## Discussion

The VHF and GPS data sets of our study cohort complement each other quite well and provide more of an enhanced understanding of winter use of habitat by deer than either data set would individually. The less sophisticated and less expensive technology of the VHF collars allowed us to collar and monitor the winter daytime locations of many deer (267 females) long-term (12 years), facilitating a more in-depth examination of population-level habitat use patterns as a function of environmental conditions (i.e., winter weather). Conversely, the more recently developed and expensive GPS collars permitted us to more continuously (daytime and nighttime) monitor the winter locations (use of habitat) of a subsample of the study cohort during a briefer part of the study period. Consequently, the breadth of environmental variability was narrower, and it was more difficult to assess temporally changing use patterns in response to weather conditions, but the finer scale temporal locations allow for a more in-depth exploration of the variability of within and among individual response patterns. Specifically, it became clear from GPS data that individuals respond differently during the same type of winter conditions, whether it be relative to use of specific habitat types (e.g., dense conifer cover) as noted here, or relative to seasonal migration or winter food habits as we’ve reported elsewhere [37], [58]. Interestingly, our observation of no apparent difference in habitat use diurnally and nocturnally by the GPS-collared deer was consistent with a recent report of no differences in diurnal and nocturnal winter home ranges or movements of GPS-collared deer in north-central MN; however, the latter study was conducted during two historically mild winters which may have tempered any potential for deer to behave differently [51]–[52].

Overall, deer increased their use of dense conifer cover as snow depths increased, and to a lesser extent, deer decreased their use of dense conifer cover as daytime temperatures decreased. Patterns were more pronounced at Wil and Shi, the sites with the highest availability of conifer cover. Together, these patterns are consistent with expectation 1B2C of Table 1, and suggest: 1) conifer cover may be limiting at Dir and Ing; and 2) conifer cover is more important as snow shelter than as thermal cover. These results are consistent with and expand on past assertions that availability of conifer cover relative to deciduous cover is a primary factor influencing where deer concentrate in winter [5], [59]–[60].

Moen estimated that the energy cost of walking through snow 53 cm deep (6 * 70 * Body weight^0.75^) for a 60-kg doe in northern MN is 1.5 and 2.0 times the cost of walking through 36 and 18 cm, respectively [61]. Increased use of dense conifers, where snow depths are much shallower [16], [18], but food is less abundant [4], [23], [58], [62], suggests a survival adaptation heavily dependent on energy conservation. Maximum snow depths of 100 cm during our long-term study had a dramatic adverse impact on the survival of collared females despite their increased use of dense cover where most available [10], [29], and severe nutritional restriction contributed directly and indirectly (e.g., surplus-killing by wolves [13], [39]) to the highest winter mortality rate (winter 1995–1996) of the study. Even within dense conifer stands, reduced maximum snow depths were deep enough (≥64 cm) to impose relatively high energetic costs for movement, except where well-worn trails may have been used. However, with time, movements restricted to such trails would limit access to otherwise available forage. Our long-term food habits data indicated that deep snow and increased use of dense conifer cover on the four sites likely restricted deer to greater use of lower quality feeding sites [58]. The influence of food availability on deer use, particularly as it interacts with use of dense cover and winter complexes can be significant [3]–[4], [6], [62]–[66]. Collectively, our data indicate that the availability or the arrangement of dense cover and higher quality feeding sites were particularly inadequate during the winter of deepest snow [10], [13], [58].

While deer also used dense conifer cover increasingly as daytime ambient temperatures increased (measured with VHF data), again, particularly on the sites where dense conifer was most available (Wil and Shi), the increasing trend relative to ambient temperature was not as dramatic as it was relative to snow depth, indicating the greater influence of the latter. Interestingly, we’ve reported that whereas snow depth had a pronounced negative impact on deer survival during this 15-year study, we could not discern any such effect of varying ambient temperatures [10], [29]. Further, the most reasonable interpretation of the trend here relative to temperature likely has more to do with the deer’s decreased use of dense cover as daytime ambient temperatures dropped below freezing (0°C), a behavioral response to adjust its critical thermal environment to benefit from increased exposure to solar radiation in the more open cover types [3], [21], [31]–[32]. Parker and Robbins [67] reported thermally critical environments for mule deer (*O. hemionus*) and elk (*Cervus elaphus*) calves in winter at operative temperatures<−20°C and >5°C; piloerection and shivering first occur at temperatures below −20°C. Similarly, the highest winter ambient temperature at which shivering occurred in white-tailed deer was −20°C [68]. These authors also found that heart rates and energy expenditure of deer increased below and above 5–10°C. Consequently, deer may benefit from reduced exposure to solar radiation when temperatures are above 10°C and benefit from increased direct exposure to sun in open habitat when temperatures are below 5°C. It is noteworthy that both of these patterns are in contrast somewhat to the long-recognized thermal cover hypothesis, which emphasizes thermal attributes of dense conifer stands and potential benefits afforded to deer by long-wave radiation when night-time temperatures are particularly low [21], [31]. On clear days, a high percentage of solar radiation is transmitted through the atmosphere; depending on its angle of incidence striking a deer’s brown pelage around its body, absorptivity can be as high as 85% and contribute significantly to their maintenance of homeothermy [32]. The greatest changes in habitat use over the wide range of ambient temperatures were relative to dense conifer cover and open types, whereas use of moderately dense cover remained low and relatively stable throughout.

Lastly, we note several important implications of our findings concerning the ability to infer the importance of conifer cover to deer relative to winter severity. Studies must be long enough to observe deer behavioral responses to winter weather conditions ranging from mild to severe [34]–[35], [69], and given the pronounced among-animal variability we observed in habitat use by our GPS-collared deer, the study cohort must be large enough to confidently assess a population-level response. Long-term studies are needed to assess the potential impact of human disturbance and alternative management actions (e.g., timber harvest operations) on survival, reproduction, habitat and space use, since demographic rates and behavioral responses often demonstrate high variability under natural conditions [22]. Further, our collective findings [58] and those of others [21], [70] suggest that deer use of conifer cover and benefits derived depend heavily on its availability and arrangement with quality feeding sites. To adequately assess habitat use and its value will require large study sites, examination of associated energetics or condition [21] and fitness of these deer relative to survival and reproductive success over time.

## Supporting Information

Figure S1 **The number of female white-tailed deer monitored by very high frequency (VHF) telemetry for habitat use on each of four study sites, north-central Minnesota, 1 November–14 May 1993–1994 to 2004–2005.**
(PDF)

Figure S2 **Distribution of sample sizes of locations for female white-tailed deer monitored by very high frequency (VHF) telemetry for habitat use (four study sites pooled), north-central Minnesota, 1 November–14 May 1993–1994 to 2004–2005.**
(PDF)
