# Correction: A Long-Term Assessment of the Variability in Winter Use of Dense Conifer Cover by Female White-Tailed Deer

**DOI:** 10.1371/journal.pone.0094893

**Published:** 2014-04-04

**Authors:** The PLOS ONE Staff

Errors occurred during the preparation of this article for publication. Equation Four of the Data Analyses section under the subheading VHF telemetry in the Methods Section is incorrect. The correct equation is *D*  =  *A_O_* + *A_B_* exp(*β*_B,0_+*β*_B,S_ *S_i,j_*+*β*_B,T_ *T_i,j_*) + *A_C_* exp (*β*_C,0_+*β*_C,S_ *S_i,j_*+*β*_C,T_ *T_i,j_*)

The publisher apologizes for these errors.
