# Correction: A Long-Term Assessment of the Variability in Winter Use of Dense Conifer Cover by Female White-Tailed Deer

**DOI:** 10.1371/journal.pone.0178964

**Published:** 2017-05-30

**Authors:** The PLOS ONE Staff

The images for Figs 1, 2 and 3 are incorrectly switched. The image that appears as Fig 1 should be should be Fig 3; The image that appears as Fig 2 should be Fig 1; The image that appears as Fig 3 should be Fig 2. The figure legends appear in the correct order. The publisher apologizes for the errors.

**Fig 1:**
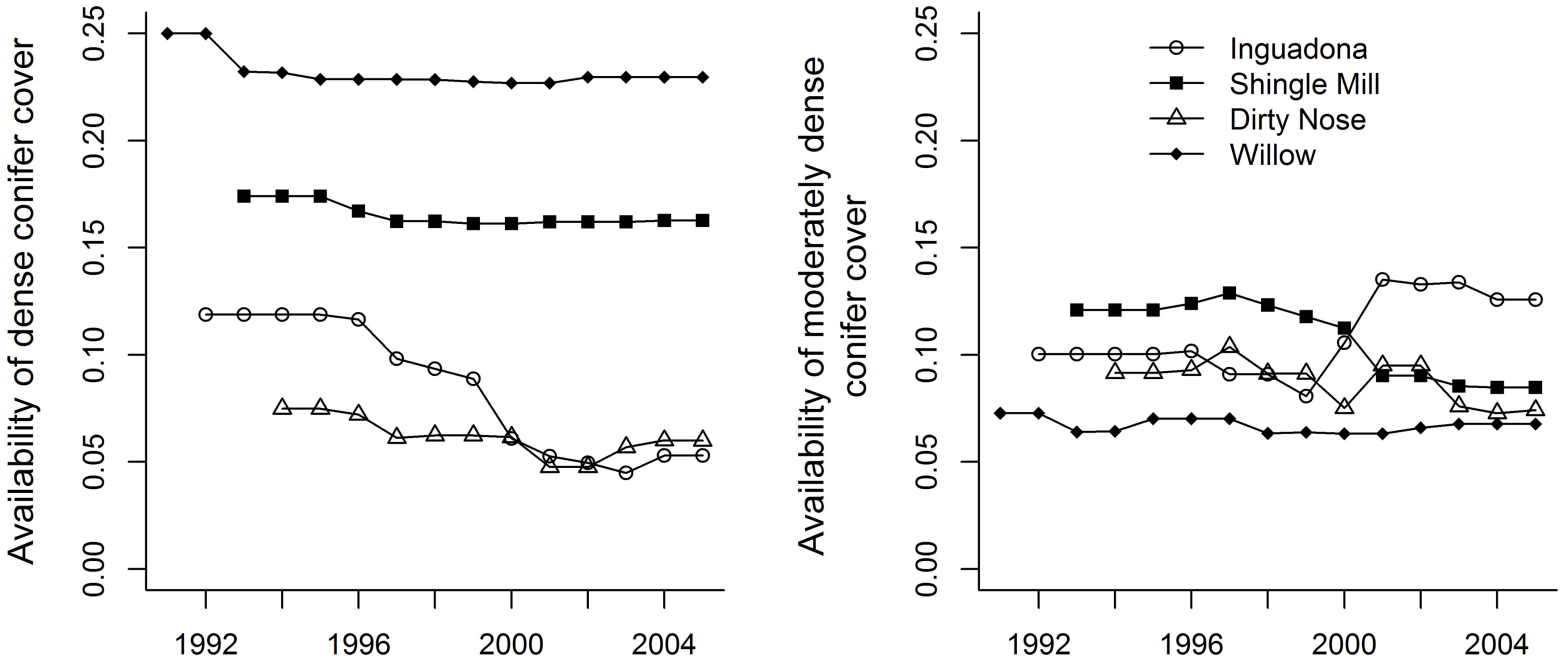
Availability (versus time) of dense (≥70% canopy closure, left panel) and moderately dense (40% ≤×<70% canopy closure, right panel) conifer cover for each of four study sites, north-central Minnesota, 1991–2005. First-year baseline was dependent on the year the site was incorporated into the study and habitat quantified.

**Fig 2:**
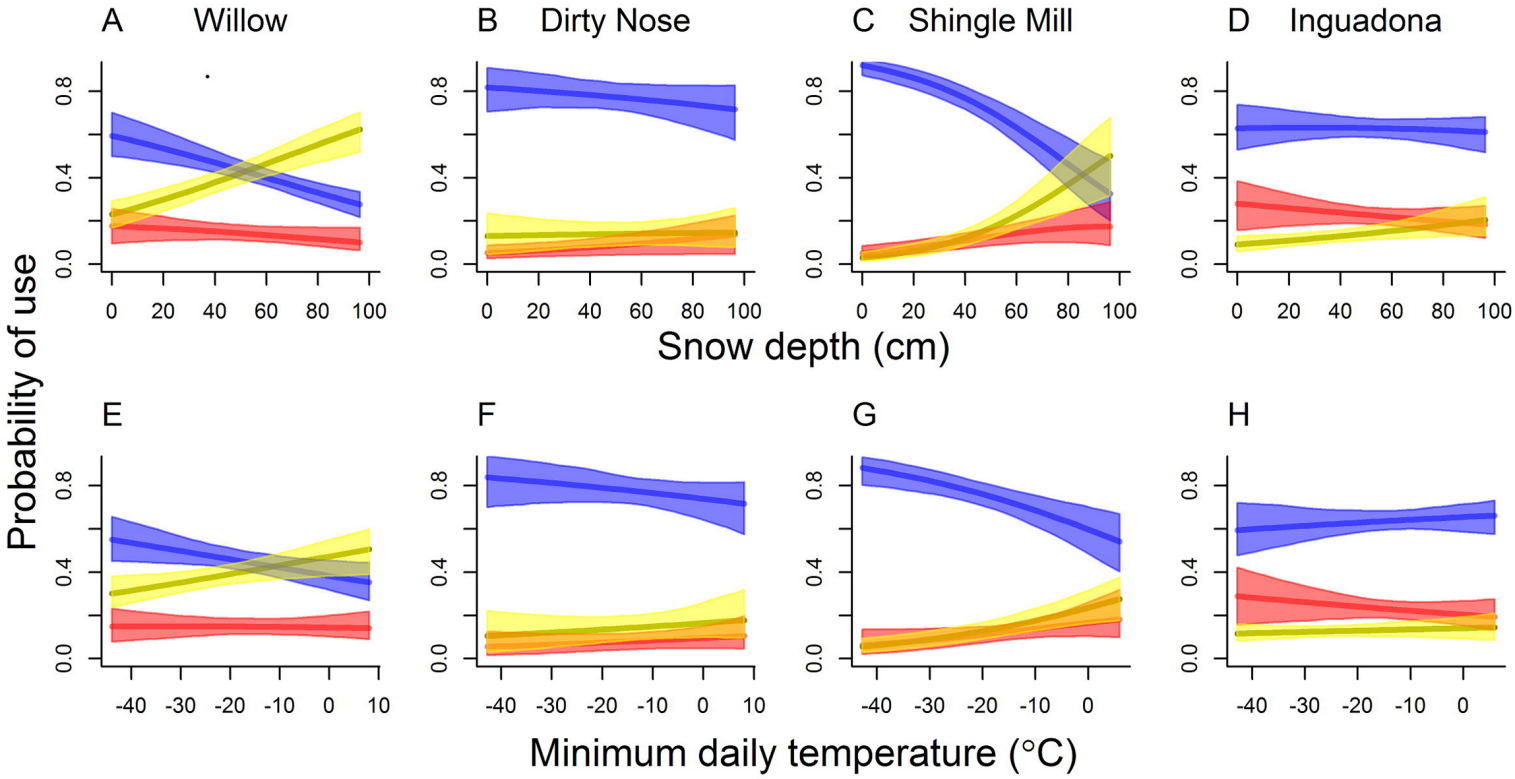
Model-based predicted probabilities of adult (≥1.5 years old), female white-tailed deer using dense (≥70% canopy closure, yellow) and moderately dense (40% ≤×<70% canopy closure, red) conifer cover, and “other” (here includes conifer with <40% canopy closure, openings, and hardwoods; blue) during daytime hours (i.e., 0730–1700 hr) as a function of snow depth (panels A-D) and minimum daily temperature (panels E-H), for each of four study sites, north-central Minnesota, 1 November–14 May 1993–1994 to 2004–2005. Colored bands depict point-wise 95% bootstrap confidence intervals. To generate model-based response curves, we set availabilities of each habitat type to site-specific mean values. Similarly, we set daily snow depths (for bottom panels) and minimum temperatures (for top panels) to site-specific means.

**Fig 3:**
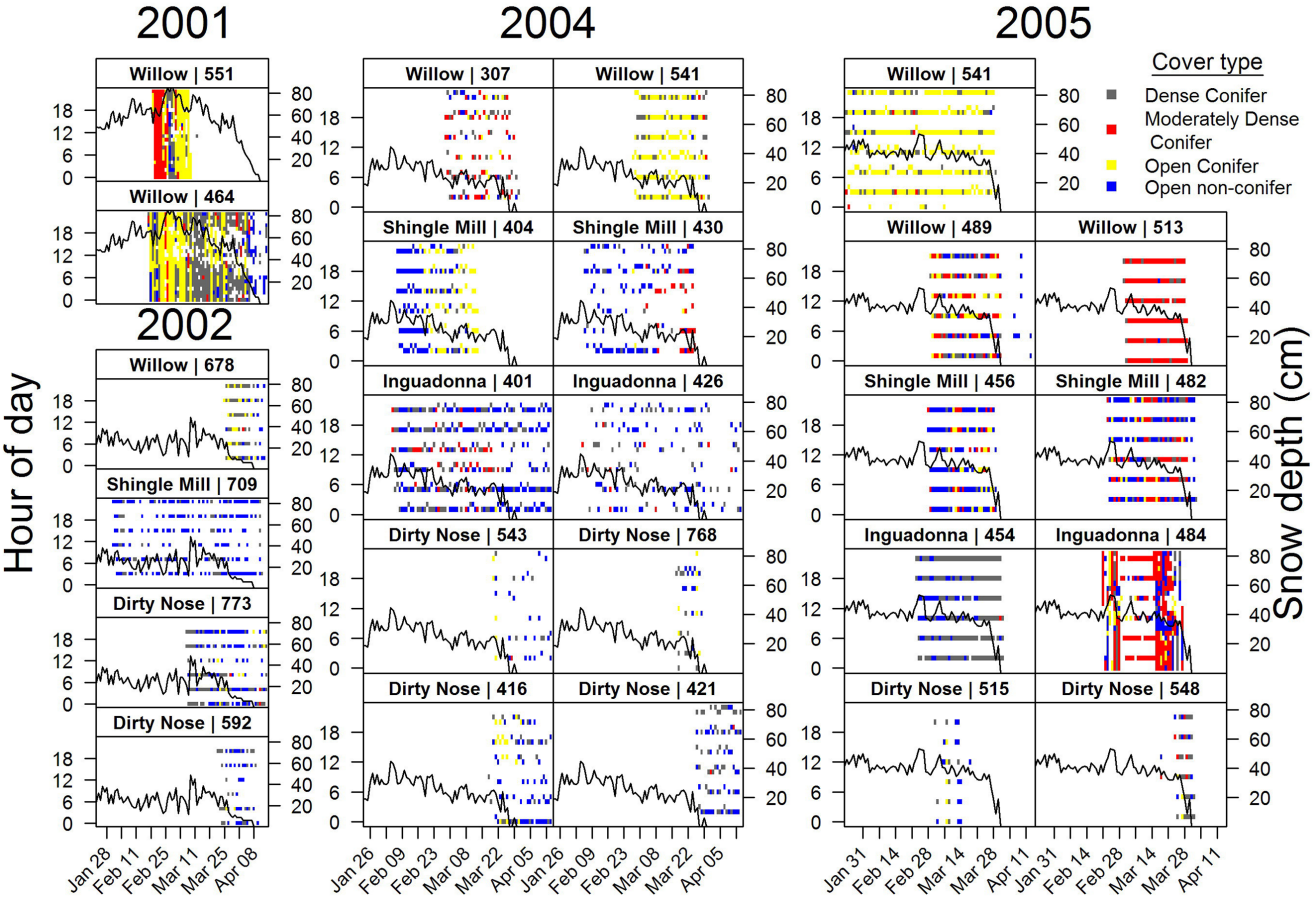
Date-time plots illustrating individual variability in use of dense (≥70% canopy closure), moderately dense (40% ≤×<70% canopy closure), and open conifer cover (<40% canopy closure), and open non-conifer types (openings and hardwood types) by adult (≥1.5 years old), female white-tailed deer monitored using Global Positioning System (GPS) collars collecting locations hourly or every four hours on four study sites, north-central Minnesota, 23 January–14 April 2001, 2002, 2004, and 2005. The solid black line represents average weekly snow depths.
