# Supplementary figures and images for: A Long-Term Assessment of the Variability in Winter Use of Dense Conifer Cover by Female White-Tailed Deer
Source: PLoS One. 2013 Jun 13;8(6):e65368. doi: 10.1371/journal.pone.0065368 (PMC3681817; doi:10.1371/journal.pone.0065368)

Number of deer

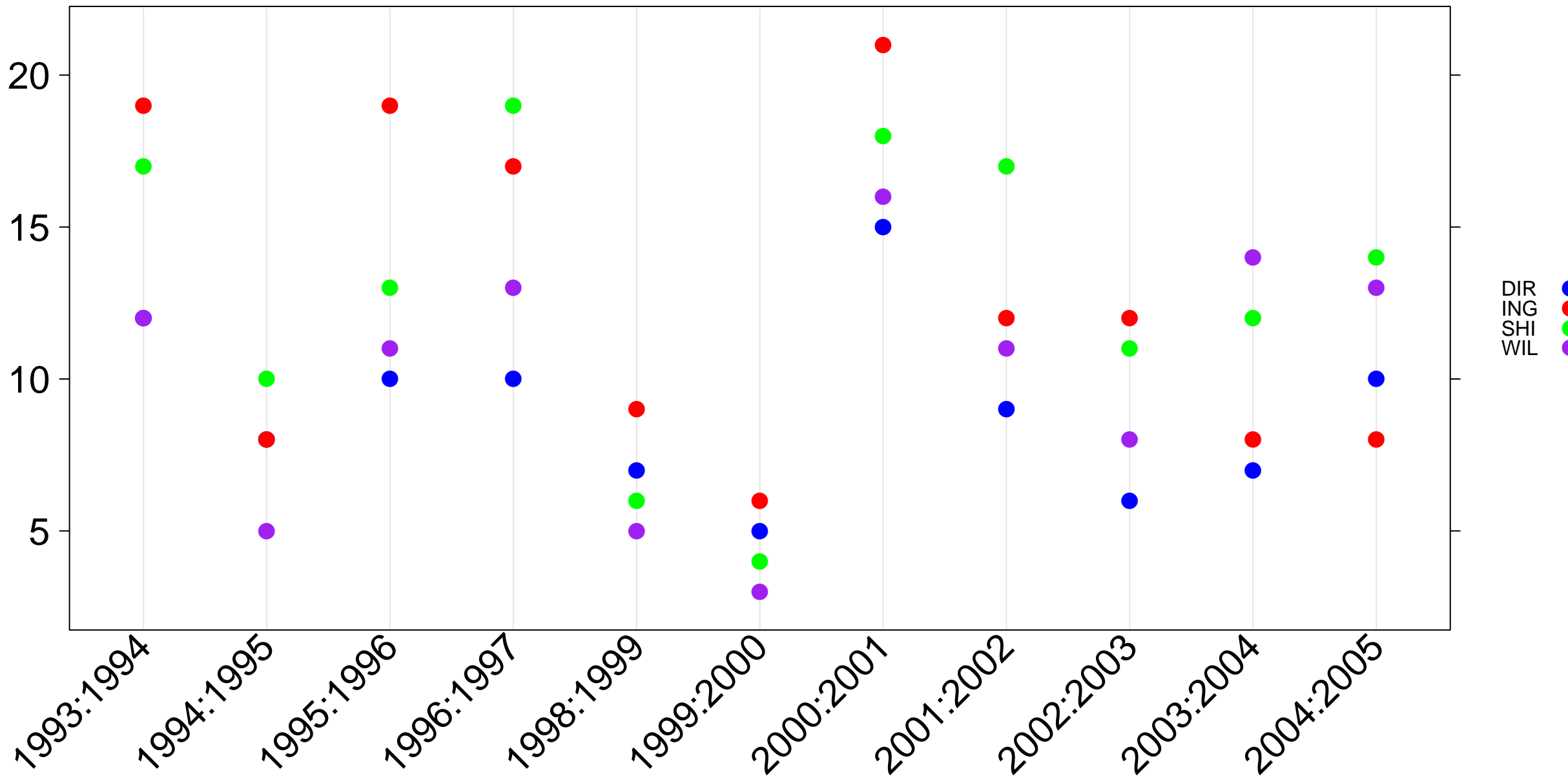

Supplement: Figure S1 — The number of female white-tailed deer monitored by very high frequency (VHF) telemetry for habitat use on each of four study sites, north-central Minnesota, 1 November–14 May 1993–1994 to 2004–2005. (PDF) [file pone.0065368.s001.pdf]

Distribution of sample sizes per deer

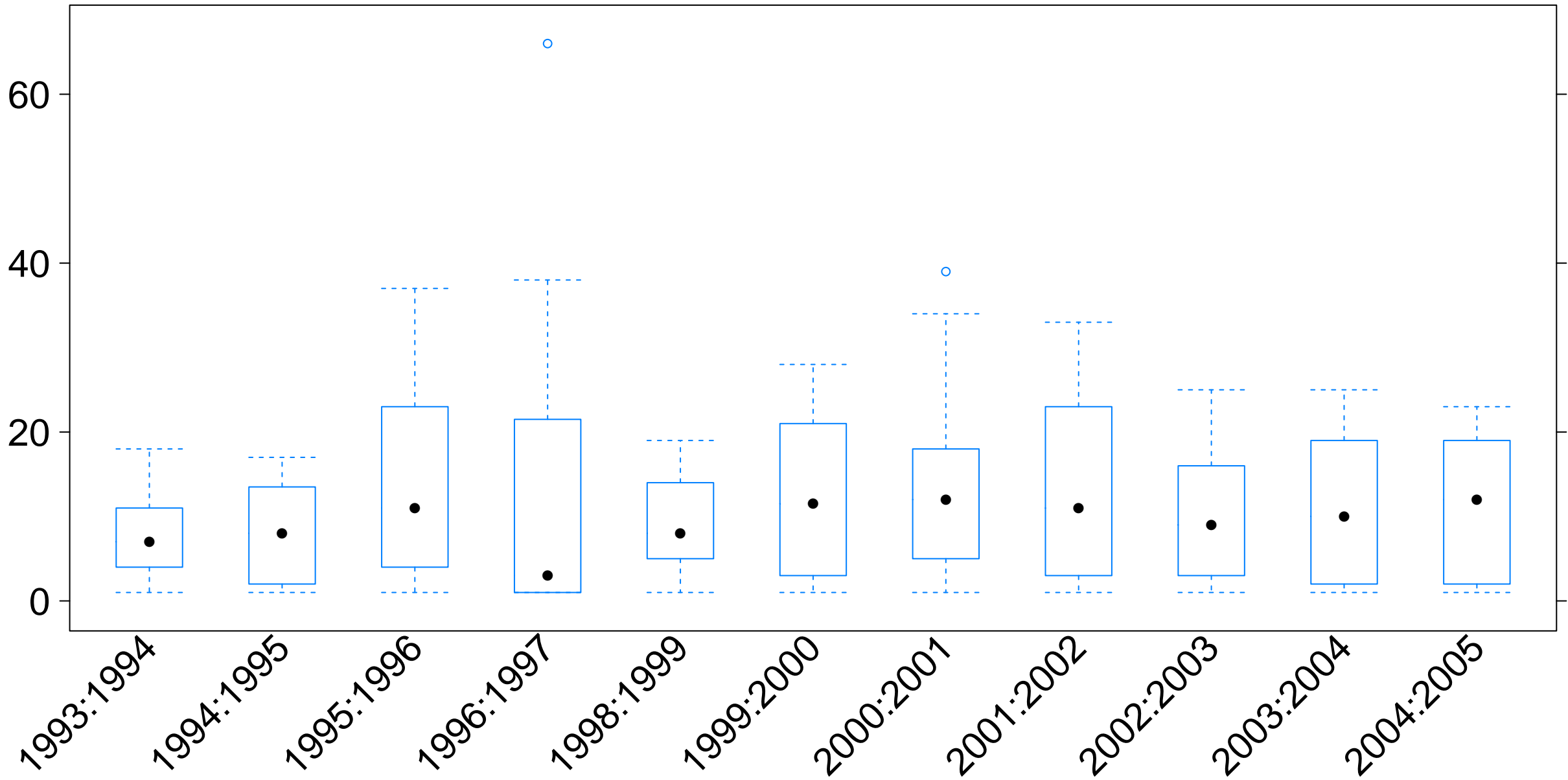

Supplement: Figure S2 — Distribution of sample sizes of locations for female white-tailed deer monitored by very high frequency (VHF) telemetry for habitat use (four study sites pooled), north-central Minnesota, 1 November–14 May 1993–1994 to 2004–2005. (PDF) [file pone.0065368.s002.pdf]
